# Representation of women in executive and academic roles within scientific societies in the field of European Endodontology

**DOI:** 10.1111/iej.14237

**Published:** 2025-04-18

**Authors:** Ana Arias, Henry F. Duncan, Ruth Perez Alfayate, Jenifer Martín‐González, Paula Riaza

**Affiliations:** ^1^ Department of Conservative and Prosthetic Dentistry, School of Dentistry Complutense University Madrid Spain; ^2^ Division of Restorative Dentistry and Periodontology Dublin Dental University Hospital, Trinity College Dublin Dublin Ireland; ^3^ Department of Clinical Dentistry, Faculty of Biomedical and Health Sciences Universidad Europea de Madrid Madrid Spain; ^4^ Department of Stomatology, Faculty of Dentistry University of Seville Seville Spain

**Keywords:** academic role, authorship, endodontology, equality, executive role, gender equity

## Abstract

**Aim:**

To explore gender distribution within the field of Endodontology in Europe and to evaluate the representation of women in executive and academic roles within scientific societies as well as national and international conferences.

**Methodology:**

After ethical approval, an online questionnaire was developed and shared with the European Society of Endodontology and the 36 national Endodontic societies officially affiliated. The survey inquired about gender composition amongst members, executive committee roles, participation in major congresses and representation in leadership and authorship positions within scientific journals affiliated with these societies over the last 5 years. Information was also retrieved from publicly available websites. Genders of the first, last and corresponding authors from scientific journals were identified. The relative ratio of women amongst society members, executive committee roles, participation in major congresses and representation in leadership and authorship positions within scientific journals was calculated. The ratio of women in leadership positions was statistically compared with the mean relative ratio of women amongst society members using a one‐sample t‐test. The current proportion of women in individual roles was compared with the general proportion of women members using the Binomial test.

**Results:**

Women account for 44% of members in endodontic societies. No significant discrepancy was observed in the overall representation of women as members in executive or editorial committees or in authorship positions within scientific journals in relation to the mean relative ratio of female society members but are significantly underrepresented in key positions such as society presidents, treasurers and scientific event organizers (*p* < .05). Additionally, women were significantly underrepresented at scientific events, both as lecturers and workshop leaders, at major congresses of national/international societies (*p* < .05). Few initiatives have been implemented to address these inequities; only three societies reported programmes aimed at promoting women in leadership or scientific roles.

**Conclusions:**

Within the limitations of this study, women appear to be fairly represented as members of executive and editorial committees in national endodontic societies and the ESE, as well as in authorship positions within scientific journals, but underrepresentation of women in positions of power and scientific forums persists. Some countries have begun implementing strategic measures to promote gender equity.

## INTRODUCTION

The representation of women in the health sector has attracted growing attention in recent years. The proportion of women working within the health sector has grown over time, specifically in higher wage healthcare occupations such as doctors, dentists and pharmacists. For example, the proportion of female physicians increased by 13% between 2000 and 2017, with an average annual growth rate of 0.58% (Boniol et al., 2019). The World Health Organization has reported that women comprised 67% of the healthcare workforce across 104 countries analysed in 2019 (https://www.who.int/publications/i/item/gender‐equity‐in‐the‐health‐workforce‐analysis‐of‐104‐countries). Moreover, in recent years, the majority of health workers in higher wage health occupations under the age of 40 are women (Boniol et al., 2019).

Likewise, the number of women enrolling in dental schools has been increasing (Furtinger et al., 2013), with more women than men graduating in recent years (Tiwari et al., 2019). By 2016, the percentage of women graduating from dental school exceeded 70% in 10 European countries (Kravitz et al., 2016). Moreover, most of these graduates expressed a desire to pursue post‐graduate studies after completion of their degrees (Pullishery et al., 2021). Despite the growing presence of women in the workforce, gender stereotypes persist from early childhood in some cultures, partly influencing career motivation, practice characteristics and opportunity (Wang & Degol, 2017). In fact, married women physicians with children worked significantly fewer hours than men in the same profession, which might directly impact their earnings and perpetuate the gender pay gap. However, addressing the issues, potential prejudices and barriers as to why women worked less could help to resolve these issues (Skinner et al., 2023). On average, female dentists also worked 3.7 hours fewer per week than men (Boniol et al., 2019) and faced more career interruptions (Kfouri et al., 2013).

Notably, the increase of women both in the dental profession and in dental schools seems not to be accompanied by a similar increase in leadership positions, as evidenced by executive and academic roles within scientific societies. Women remain poorly represented in professional organizations (Campus et al., 2024) and are underrepresented in leadership positions across all sectors globally (Gangwani et al., 2024; Garcia et al., 2022; Tiwari et al., 2019). Within general dentistry, the representation of women in academic positions and leadership roles has seen some progress, yet significant disparities remain. In fact, women seem to be well represented at entry‐level academic and professional positions, but their presence decreases as ranks advance towards the top. Studies indicate that whilst women constitute approximately 36% to 40% of dental faculty, their presence in higher academic ranks, such as professors, is disproportionately low, ranging from 18% to 26% (Kim et al., 2024). Only 18% of all dental schools in the United States of America (USA) had female deans in 2021 (Bompolaki et al., 2022). Likewise, most full‐time faculty positions (59.5%) at dental schools in the United States were held by men who were also compensated financially at higher rates than their female counterparts, with female deans making 7% less money in wages and add‐ons (Garcia et al., 2022). Researcher roles show similar figures. Women tend to be significantly underrepresented in research both as researchers and research participants, receive less research funding, and appear less frequently than men as authors on research publications (Ovseiko et al., 2016). When the dental literature indexed in Thomson Reuters Web of Knowledge Journal Citation Reports was analysed in 2012, only 14.8% of editorial board members across the 69 major dental journals were women (Ioannidou & Rosania, 2015). If publications of the 10 journals with the highest impact factors in the field of dentistry (Journal Citation Reports in 2016) in their dental field were assessed (Sartori et al., 2021), the inequalities in scientific production between genders increased in positions of greater prestige or leadership. The prevalence of women as first authors was 37.2% and decreased as last authors to 22.6%. At the same time, the presence of a woman as the last author increased the presence of women in the first author position by 16% (Sartori et al., 2021). Recent studies have reported the same trends, that women represented 38% of dental researchers and only 15% of dental journal editors (Bennie & Koka, 2021; Tiwari et al., 2019).

Similarly, gender inequality has also been reported from several countries relating to speakers in dental meetings. In Spain, 80% of national and international speakers in scientific meetings were men (Hernández‐Ruiz et al., 2022). In the United Kingdom, only three specialties (paediatric dentistry, endodontics and general public health) had an acceptable balance of male/female speakers in their annual meetings (>40%) in 2018/2019 (Heggie et al., 2021). In Italy, out of all specialties, except for paediatric and special care dentistry, women were significantly underrepresented, specifically in the field of endodontics (female/male representation: 48/315 from 2019 to 2022) (Bernardi et al., 2023).

Some challenges and barriers have been described as an explanation for this underrepresentation. Gender inequities seem to be related to difficulties in work–life balance, family and societal pressures, lack of role models, mentors and leadership training opportunities, limited access to research funding and intentional or unintentional gender bias (Gangwani et al., 2024; Tiwari et al., 2019).

However, research has demonstrated that increasing women's participation leads to significant benefits across various sectors. These benefits include economic growth, improved work quality, greater innovation and higher employee satisfaction (Gangwani et al., 2024). In academia, studies have shown that scientific articles with mixed‐gender authorship tend to receive higher citation rates (Bennie & Koka, 2021). Recent studies highlight that gender inequities are not only detrimental to individual careers but also negatively affect the broader professional and scientific landscape (Glick & Fiske, 2011). Moreover, gender bias is identified as a key factor contributing to job dissatisfaction, leading to a higher abandonment rate amongst women in academic positions (Kim et al., 2024). Recognizing these challenges, the World Health Organization has called for the continuation and implementation of targeted policies to promote gender equity in the healthcare sector (https://iris.who.int/bitstream/handle/10665/311314/WHO‐HIS‐HWF‐Gender‐WP1‐2019.1‐eng.pdf?sequence=1).

Although several studies have addressed and studied the gender inequity in dentistry, there is a notable paucity of data analysing the representation of women in leadership and recognition roles in specialized fields including endodontics (Gangwani et al., 2024). Investigating gender distribution in dentistry, and specifically within endodontics, is essential for developing evidence‐based policies that target this issue.

Therefore, the aim of this study was to analyse the gender distribution within the field of endodontology in Europe, and to evaluate the representation of women in executive and academic roles within professional and scientific societies as well as national and international conferences.

## METHODOLOGY

### Ethical approval

The current study was conducted with the approval of the Ethics Committee of Complutense University of Madrid (CE_20241010_29_SAL) adhering to the Organic Law on Data Protection and Digital Rights Guarantee (LOPDGDD 3/2018 of December 5).

### Study design

A retrospective longitudinal study was conducted to assess the current representation of women in Endodontology in Europe and its evolution over the past 5 years (2020–2024).

### Study population

An online questionnaire was developed via Google Forms and shared with the 36 national Endodontic society members officially affiliated with the European Society of Endodontology (ESE), as well as with the ESE itself.

The project leader (AA) contacted the country representatives for each national society and the ESE executive board twice and kindly asked them to voluntarily complete a survey, thereby consenting to the integration of the responses for subsequent aggregated analysis and reporting.

### Data collection from questionnaires

The survey was designed to gather detailed information on gender representation within scientific societies over the last years (since 2020). It focused on gender composition amongst members, executive committee roles, participation in major congresses and representation in leadership and authorship positions within scientific journals affiliated with these societies.

**FIGURE 1:**
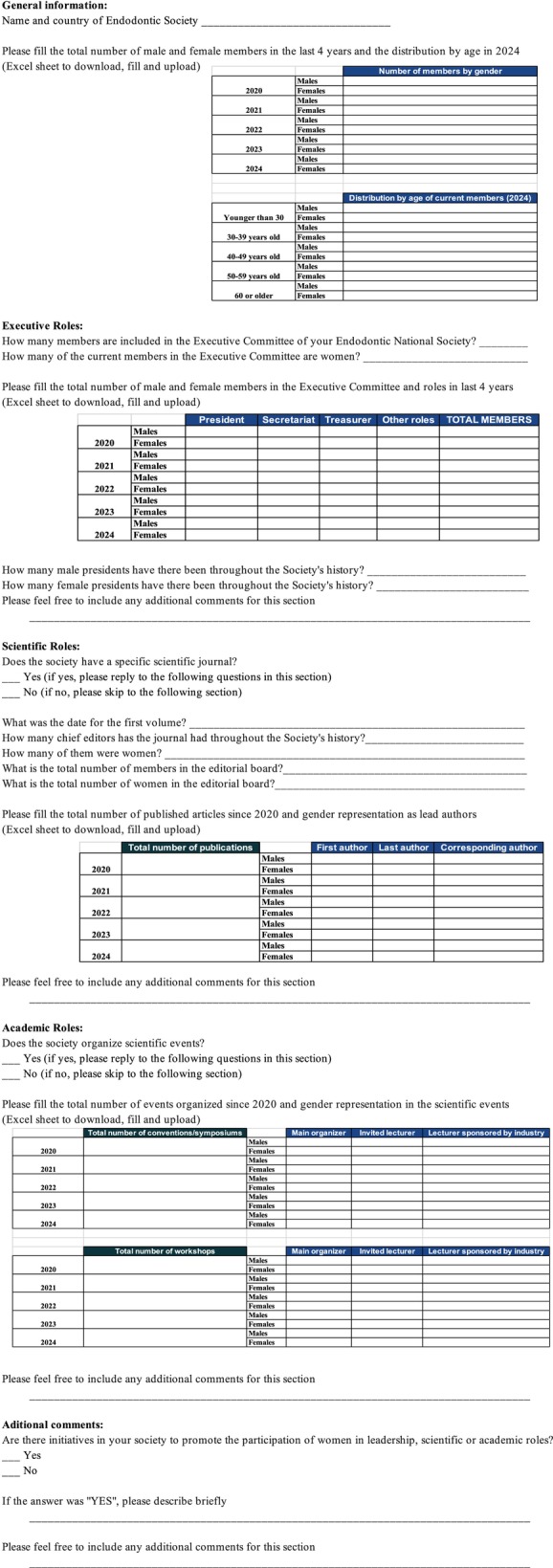
Online questionnaire.

The survey (Figure 1) comprised five main sections

1. *General information*: Gender and age distribution of society members.
2. *Executive roles*: Gender representation in executive committees.
3. *Scientific roles*: Gender distribution amongst chief editors of scientific journals, editorial board members and authorship positions (first, last and corresponding authors) for societies with affiliated journals.
4. *Academic roles*: Gender representation amongst lead organizers, lecturers and workshop leaders for societies organizing scientific events.
5. *Gender equity initiatives*: Information on initiatives aimed at promoting women's participation in leadership, scientific and academic roles.

The survey employed various question formats, including open‐ended questions requesting short numerical or categorical responses, close‐ended binary questions (yes/no) and open‐ended questions inviting feedback. Four spreadsheets were provided for societies to record data on gender representation over the past 5 years. These spreadsheets detailed the gender distribution of members affiliated with the society, executive committee members, editorial board members of their journal (if applicable) and participants in scientific meetings.

Data from those societies completing the online questionnaire were collected in Excel spreadsheets.

### Data collection from the Internet

For society representatives that did not respond to the questionnaire, publicly available data from their official websites was sourced through links provided on the ESE website. If a link was unavailable, a web search was conducted to locate the official websites of the scientific societies. For webpages in languages other than Spanish or English, Google Translate was used for translation.

All accessible information from the past 5 years was collected and recorded, including

- The total number of presidents in the society's history and the total number of female presidents.
- The gender of key executive committee members, such as the chair/president, secretary, treasurer and other board members.
- The presence of a linked scientific journal, along with the total number of chief editors, the number of female chief editors, the total number of editorial board members, the number of women on the editorial board and authorship of their publications.

In addition, an internet search was conducted to analyse conference programmes from the past 5 years, aiming to determine the gender of speakers. Programmes were accessed via official society websites as well as other platforms such as Facebook or Google Images.

### Data collection from the international endodontic journal

A search was conducted in two principal databases, PubMed and Web of Science, to retrieve all articles published in the *International Endodontic Journal* over the last 5 years, from January 2020 to November 2024. After removing duplicates, the names and genders of the first, last and corresponding authors for each article were recorded. Gender was recorded as a binary categorical variable (woman/man). When the gender was not readily identifiable by name, a manual search for public photographs, university profiles was performed following methods described in previous studies (Bennie & Koka, 2021; Bompolaki et al., 2022; Hernández‐Ruiz et al., 2022). Sources used included university or affiliated organization websites, ResearchGate profiles and ORCID profiles. If gender was not identified with the previous methods, a further search was accomplished in www.genderize.io.

### Statistical analysis

Data analysis was conducted using SPSS Statistics Version 29.0 (IBM Corporation; Armonk, NY, USA). A descriptive analysis was performed to calculate the relative ratio of women amongst society members, executive committee roles, participation in major congresses and representation in leadership and authorship positions within scientific journals. The ratio was calculated for each year independently. An inferential statistical analysis was conducted afterwards. For those variables for which historical information could not be retrieved, the analysis was performed only for current data. In a first set of analysis and after confirmation of the compatibility of data with a normal distribution, the ratio of women in leadership positions for each specific year was statistically compared with the mean relative ratio of women amongst society members for that same exact year (which served as a reference value) using the one sample *t*‐test and 95% confidence intervals (CIs) were calculated. The current proportion of women and men in individual roles of the executive committees (president, secretary, treasurer) was compared with the general proportion of women and men society members using the Binomial test. Statistical significance was set at *p* < .05.

## RESULTS

Ten national societies (out of 36) and the ESE (11 in total) replied to the questionnaire via Google Forms or email, whilst additional information for 21 other national societies was retrieved from their official websites or other online platforms. Finally, five countries did not respond, and the information could not be retrieved from their websites. Membership data were available only from the societies that responded via Google forms.

Amongst the 11 responding societies, all of them organized scientific events, with nine providing data on gender distribution amongst lecturers participating in these events. Only two societies included or disclosed data on sponsored lectures, limiting the analysis of this parameter to descriptive statistics. Additionally, five societies provided information on workshops held during their scientific events. Three societies reported having a specific scientific journal, although one had not published any manuscripts in the last 2 years. Furthermore, three national societies had implemented initiatives to promote the participation of women in leadership, scientific or academic roles.

For the 21 societies whose data were obtained online, membership information, including general gender distribution, was unavailable. However, data on the current composition of their executive committees were accessible. Scientific programmes in major congresses could be retrieved from 12 national societies and general data for gender distribution amongst lecturers were registered. Four of these societies had a specific scientific journal, but publications could not be accessed online to search the gender distribution for the different leadership author positions and only one of them provided publicly accessible data on its current editorial committee.

Table 1 shows the relative ratios of women (mean, standard deviations and minimum and maximum values; as well as the number of national societies represented by those figures) for general membership and the different executive, academic and scientific roles over the past 5 years.

**TABLE 1:** Relative ratios of women (mean, standard deviations and minimum and maximum values; as well as the number of national societies represented by those figures), for general membership and the different executive, academic and scientific roles over the past 5 years.

	Role	Year	Ratio (*n* women/*n* total)				Valid responses
			Mean	SD	Min	Max	*n*
General information	Society members	2020	0.45	0.19	0.17	0.65	6
		2021	0.46	0.19	0.17	0.64	6
		2022	0.46	0.19	0.17	0.64	6
		2023	0.45	0.17	0.18	0.64	6
		2024	0.44	0.16	0.17	0.63	8
Academic role	Main organizer	Total (2020–2024)	0.25	0.20	0	0.5	7
	Lecturers	2020	0.17	0.22	0	0.67	8
		2021	0.14	0.13	0	0.33	6
		2022	0.24	0.17	0	0.53	12
		2023	0.23	0.13	0	0.43	11
		2024	0.22	0.17	0	0.5	22
	Sponsored lecturers	2020	0.32	.25	0.14	0.5	2
		2021	0.25	.35	0	0.5	2
		2022	0.23	.24	0.08	0.5	3
		2023	0.28	.20	0.10	0.5	3
		2024	0.5		0.5	0.5	1
	Workshops	Total (2020–2024)	0.15	.13	0	0.34	5
Scientific role	Editor	Total (historical)	0.22	0.38	0	0.67	3
	Editorial board	Current	0.23	0.26	0	0.60	3
	First author	2020	0.66	0.31	0.39	1	3
		2021	0.56	0.27	0.39	0.88	3
		2022	0.54	0.17	0.38	0.72	3
		2023	0.61	0.19	0.48	0.75	2
		2024	0.53	0.19	0.39	0.67	2
	Last author	2020	0.54	0.40	0.29	1	3
		2021	0.26	0.23	0	0.42	3
		2022	0.50	0.21	0.26	0.64	3
		2023	0.52	0.18	0.39	0.65	2
		2024	0.43	0.04	0.4	0.45	2
	Corresponding author	2020	0.50	0.44	0.17	1	3
		2021	0.26	0.23	0	0.44	3
		2022	0.52	0.24	0.27	0.75	3
		2023	0.54	0.23	0.38	0.71	2
		2024	0.46	0.06	0.42	0.50	2

	Role	Year	Ratio (*n* women/*n* total)				Valid responses
							*n*
Executive role	President	2020	0.29				14
		2021	0.33				15
		2022	0.33				15
		2023	0.25				16
		2024	0.26				31
Executive role	Secretary	2020	0.3				10
		2021	0.27				11
		2022	0.27				11
		2023	0.27				11
		2024	0.33				24
	Treasurer	2020	0.27				11
		2021	0.25				12
		2022	0.33				12
		2023	0.33				12
		2024	0.29				24
			**Ratio (*n* women/*n* total)**				**Valid responses**
			**Mean**	**SD**	**Min**	**Max**	***n***
	Other	2020	0.49	0.34	0	1	12
		2021	0.49	0.30	0	1	13
		2022	0.44	0.32	0	1	13
		2023	0.42	0.30	0	1	13
		2024	0.36	0.30	0	1	27
	Total	2020	0.40	0.23	0	0.80	13
		2021	0.39	0.21	0	0.80	14
		2022	0.39	0.22	0	0.80	14
		2023	0.40	0.23	0	0.83	15
		2024	0.38	0.26	0	0.83	29
	Historical presidency		0.17	0.25	0	1	15

The mean ratio of female members in endodontic national societies is currently 0.44 (standard deviation (SD) = 0.16) with the highest representation observed in Turkey and Spain, where the ratio surpasses 0.60. The lowest representation was observed in the rates of specialist and certified members of the ESE, where the ratio of women to total members did not even reach 0.20 (ratio = 0.17). The ratio remained relatively stable over time, showing no significant changes from 2020 to present.

In a first set of inferential analysis, when the ratio of women in leadership positions and authorship roles for each specific year was compared with the mean relative ratio of women amongst society members for that same year, no significant discrepancy was observed in the overall representation of women as members in executive or editorial committees or in authorship positions (first, last or corresponding authors) within scientific journals in relation to the mean relative ratio of female society members (*p* > .05). However, comparison of the current proportion of women and men in individual roles of the executive and academic committees to the general gender proportion of members demonstrated significant discrepancies in the representation of women for most of the individual leadership roles (*p* < .05). There were no significant differences in the representation of women as secretaries of the national societies, but in general, women were significantly underrepresented in the rest of the key roles, such as president and treasurer in executive committees (*p* < .05), as well as lead organizers of scientific events (*p* < .05). Conversely, no significant discrepancies were observed for the roles of secretary or chief editor of scientific journals, although most journals have never had a female chief editor in their history. Moreover, the historical representation of male presidents in endodontic societies significantly exceeded the mean relative ratio of female members (*p* < .0001); with a 95% CI −0.013 to −0.41. Additionally, women were significantly underrepresented at scientific events, both as lecturers (*p* < .005) and workshop leaders (*p* = .004) at major congresses of the national societies. Responses to open‐ended questions providing feedback addressed the commitment of some national societies to promoting the inclusion of women in decision‐making roles, whilst others acknowledged the absence of records for historical data.

## DISCUSSION

This study aimed to evaluate the gender distribution within the field of endodontology in Europe and assess if women are fairly represented in executive and academic roles within endodontic scientific societies, as well as national and international conferences. A retrospective, longitudinal study design was adopted trying to retrieve historical records from endodontic societies and observe changes over time for a 5‐year period. The study combined multiple data sources for data retrieval to enhance reliability, in a similar method to previous studies (Hernández‐Ruiz et al., 2022; Tiwari et al., 2019); however, the current study went further as most available reports have relied solely on website information (Bennie & Koka, 2021; Bernardi et al., 2023; Bompolaki et al., 2022; Kim et al., 2024; Li et al., 2019). Data collection prioritized direct responses from the ESE and the 36 affiliated national endodontic societies via a questionnaire distributed to their representatives. The survey intended to collect information on the gender distribution amongst members and representation of women in executive committee positions, leadership roles within scientific journals, authorship of scientific publications, organizational roles in scientific events and participation as speakers in major congresses between 2020 and 2024.

When responses were not received, publicly available information from society websites, official records and congress programmes were manually retrieved. The study also included data from three scientific journals, with the *International Endodontic Journal* information being reviewed manually. According to the American Psychological Association (APA), gender refers to the societal and cultural roles, behaviours and attributes associated with being male or female (https://apastyle.apa.org/style‐grammar‐guidelines/bias‐free‐language/gender). In scientific publishing, the absence of explicit gender information for authors necessitates a subjective classification based on names and photographs to infer gender for analysis. Gender categorization was conducted through online searches for photographs, following previously established protocols or online profiles (Bennie & Koka, 2021; Bompolaki et al., 2022; Hernández‐Ruiz et al., 2022). Automated tools supplemented manual categorization when required, although it is accepted that these tools may introduce potential biases due to misclassification based on name patterns, which could affect the accuracy of gender determination. Out of 739 scientific articles published from 2020 to 2024, the gender of the first, last and/or corresponding author was unidentified in 10 (<1.4%). The unidentified <2% omission seems unlikely to impact overall findings significantly. However, despite efforts to enhance accuracy and reduce bias, this method carries inherent limitations due to cultural variations in naming conventions, potential misclassification and the inability to account for gender identity beyond binary definitions. It is recognized that this limitation might contribute to cisgender bias by assuming an alignment between gender identity and appearance, making it a significant constraint of the current study methodology.

Information regarding current executive committees' composition and recent scientific events was predominantly accessible online, whilst historical data from previous years were limited. General membership distribution by gender could not be retrieved online, and therefore, this information was based exclusively on the societies that responded to the questionnaire and were able to provide the requested data (Table 1 shows the final sample size for each time period). Also, some society representatives completing the online survey reported difficulties in accessing historical membership data, with only six societies providing retrospective data spanning the 5 years under review. For those variables for which historical information could not be retrieved, the analysis was performed only for current data.

The key limitation of the current study was the low survey response rate (11 out of 36 societies), which needed reliance on incomplete and potentially outdated information from society websites, particularly for historical data. Despite multiple attempts, no information could be retrieved from five national societies. However, the limited response rate in this study could suggest either a lack of interest in the subject of this questionnaire or alternatively could indicate that some national societies do not systematically collect gender‐related data. Therefore, the scarcity of data might be due in part to societies not maintaining records related to gender, age and other factors, rather than an unwillingness to provide the information. Indeed, another limitation of the study is that the questionnaire did not ask for a description of the type of data that societies retained. The reply to this inquiry would have helped to understand better the reasons for nonresponse and to avoid subsequent speculation. Moreover, some countries might have data protection regulations regarding the registration of specific personal data, like age and gender, that could be collected by societies. Furthermore, some regulations might request the deletion of personal data for members who leave the society; hence historical data would not be available in these societies.

Fortunately, sufficient online data were available and could be retrieved to evaluate the current representation of women in endodontics. Similarly, data from previous congress programmes were not always available, as many societies lacked accessible archives for events held in the past 5 years. Additionally, data on the current age‐specific gender distribution amongst members were requested through one of the Excel spreadsheets attached to the questionnaire; however, this information was limited, with only three societies providing responses. These factors limited the assessment of temporal trends and member demographics by age. Nonetheless, sufficient data were collected to assess the current state of women's representation in executive and academic roles across European endodontic societies, although the findings might not fully capture the complete gender distribution within the field. Despite these limitations, the primary strength of the study lies in its comprehensive approach to gender representation. Although the findings should be interpreted with caution, they still provide a meaningful snapshot of the current state of women's representation in executive and academic roles in european endodontology. By examining multiple dimensions, including professional, executive, scientific and academic roles, it provides a holistic understanding of women's representation within the field of European endodontology over the last 5 years.

Women constituted 44% of the members of the screened endodontic societies, a proportion below that of dental graduates reported in Europe between 2014 and 2016 (Tiwari et al., 2019), although in line with the proportion of enrolled female students in endodontic programmes in the United States in 2020 (Garcia et al., 2022). This discrepancy could be influenced by gender preferences for specific dental specialties. When fourth‐year dental students were inquired, 15% of male students planned to specialize in endodontics, compared to only 5% of female students (Khan et al., 2020). Previous studies also highlighted how women preferred to pursue specialty training in special care, paediatric dentistry or orthodontics, and men tended to be more inclined towards specialties like oral surgery, prosthodontics and restorative dentistry (Gallagher & Scambler, 2021; Khan et al., 2020; Surdu et al., 2021).

No significant differences were observed between women's representation in executive roles and scientific committees and their proportion as society members. These results contrast with earlier evidence indicating underrepresentation of women in these areas (Gangwani et al., 2024; Hernández‐Ruiz et al., 2022; Tiwari et al., 2019). For instance, a previous cross‐sectional study based on online data found that women occupied 28% of leadership roles in dental associations in the United States and 34.9% in Canada (Li et al., 2019), both lower than the approximately 40% observed in the present study. Similarly, Bernardi et al. (2023) highlighted significant gender disparities in national and local dental board roles, with women occupying only 15.82% of positions compared to 84.18% by men. The higher rates observed in the present study could suggest increased incorporation of women into executive roles in recent years, particularly in Europe and the field of endodontology.

Despite the overall feminization of executive committees, global studies consistently demonstrate gender imbalances in top leadership positions. For example, in Spain, female representation on the scientific society boards varied widely depending on the specialty, from 0% to 80% (Hernández‐Ruiz et al., 2022). However, over 70% of presidents and vice presidents in professional associations and more than 60% in scientific societies were men, and no women have historically held leadership roles in the general council. Similarly, in prosthodontics, 20% of European association presidents were women, whilst the American prosthodontic society had no female presidents between 2000 and 2019 (Phasuk et al., 2021). Further analysis of specific roles in the present study revealed that women were less likely to hold top leadership positions, such as president or treasurer in executive committees, but were more frequently represented in roles like secretary or other committee positions. Consistent with prior research (Bompolaki et al., 2022; Kim et al., 2024), these findings suggest that whilst women are well represented within endodontic professional societies, they generally tend to occupy lower ranked positions.

These findings highlight the challenges women face in accessing leadership positions, which may stem from several factors, including a lower number of women applying for these roles. Several authors (Bompolaki et al., 2022; Brown et al., 2020; Gangwani et al., 2024; Garcia et al., 2022; Hernández‐Ruiz et al., 2022; Kim et al., 2024) have suggested that women often encounter difficulties balancing work and personal life. Unlike men, women in leadership positions are less likely to be married or have children (Pallavi & Rajkumar, 2011). In addition, leadership stereotypes are frequently characterized by traditionally masculine attributes (Tremmel & Wahl, 2023) with terms like ‘brilliance’ and ‘genius’ more commonly associated with men (Storage et al., 2020). Women who exhibit traits typically associated with effective leaders, such as dominance, persistence and competence, are often perceived as less likable (Eichenauer et al., 2022). These biases may contribute to perceptions of women being less suitable for top leadership roles, further compounding the barriers they face in attaining such positions.

Likewise, although (Ioannidou & Rosania, 2015) reported that only 14.8% of editorial board members across 69 major dental journals were women, certain dental specialties demonstrated relatively higher proportions of women on their editorial boards compared with others. In the present study, no significant discrepancies were observed for the roles of chief editor in national scientific endodontic journals. However, the Q1‐ranked *International Endodontic Journal* has never had a female chief editor. Globally, only 15% of chief editor positions in leading dental journals are occupied by women (Bennie & Koka, 2021).

A previous study (Sartori et al., 2021) analysed leadership authorship positions in the dental journals with the highest impact factors and found that out of 3862 articles reviewed, male researchers dominated authorship position overall (68.3%), with women unrepresented as first (37.2%) and last authors (22.6%) in 2006. Between 2006 and 2016, women's representation increased by 13.2% in first authorship and 61% in last authorship. Interestingly, having a female as the last author increased the likelihood of a female first author by 16%. In contrast, the present study found no differences when analysing scientific authorship roles. Over the last 5 years, women accounted for 53–66% of first authors, 26–54% of last authors and 26–54% of corresponding authors. These figures represent an increase from 2019 when women comprised only 30% of european researchers across all dental specialties (Tiwari et al., 2019).

A key finding of the present study was the significant disparity between men and women serving as lecturers at major national and international conventions. Over the past 5 years, women have delivered only 14–24% of lectures in endodontic scientific meetings, a figure markedly lower than their representation in the field. Numerous studies have emphasized the underrepresentation of female speakers in dentistry, with female participation varying across dental specialties and geographic regions (Gangwani et al., 2024; Hernández‐Ruiz et al., 2022; Tiwari et al., 2019). In the field of Endodontology, the results of the present study, unlike other factors, indicate little progress over the last 5 years. Alarming figures have also been reported in other fields; for example, congresses with a higher number of attendees in Spain correspond to periodontics and prosthodontics and had fewer than 10% female speakers in 2019 (Hernández‐Ruiz et al., 2022). Likewise, in the field of prosthodontics, women accounted for only 10.8% of speakers at scientific meetings organized by six different prosthodontic organizations in the United States over a 10‐year period (Phasuk et al., 2021). In contrast, some dental congresses have reported higher female representation. For instance, Australian dental congresses featured 25–36% female speakers (Silva & Teoh, 2021), whilst the British Dental Association reached 39.8% female representation (Heggie et al., 2021).

The discrepancy between the proportion of women researchers and their representation as invited lecturers at scientific meetings is noteworthy. Despite increasing representation of women in leadership authorship roles in scientific publications, this has not translated into a proportional increase in female speakers. This phenomenon can partly be explained by the Matilda effect, which posits that women's contributions are often under‐recognized or acknowledged later in their careers compared to their male peers (Rossiter, 1993). Additionally, men are more likely to achieve professional recognition earlier in their careers (Giannobile & Feine, 2019).

Lastly, the fact that only three scientific societies reported implementing specific initiatives to promote women's participation highlights a significant gap in active efforts to address gender inequity. This lack of targeted measures may partially explain the persistent underrepresentation of women in leadership roles and as speakers, as well as the limited progress reflected in several of the findings of the current study.

In conclusion, whilst women appear to be fairly represented as members of executive and editorial committees in both national endodontic societies and in the European Society of Endodontology, as well as in authorship positions within scientific journals, significant underrepresentation persists in key individual roles. These include positions such as president and treasurer in executive committees, as well as lead organizers of scientific events, lecturers and workshop leaders at major congresses in the field of endodontology. Although these findings should be interpreted with caution due to the limited dataset, some countries have begun implementing strategic measures to promote gender equity. These efforts might overcome barriers such as work‐life balance challenges, leadership stereotypes, and unconscious biases that likely contribute to these disparities. Additionally, this study has highlighted a need for improved record‐keeping and data accessibility whilst respecting the limits of European and national regulations. If national societies implement systematic data collection and record keeping related to gender representation over time, future research on the topic could be enhanced, whilst also increasing transparency on gender balance and other diversity trends within professional organizations. Further efforts and targeted initiatives are necessary to continue advancing gender equality in leadership roles within endodontology.

## AUTHOR CONTRIBUTIONS

Conceptualization, A.A., H.F.D.; methodology, A.A., P.R.; formal analysis, A.A.; investigation, A.A., P.R.; data curation, A.A.; writing—original draft preparation, A.A., R.P.A., J.M.G., P.R.; writing—review and editing, A.A., H.F.D.; supervision, A.A. All authors have read and agreed to the published version of the manuscript.

## CONFLICT OF INTEREST STATEMENT

There is no conflict of interest related to this investigation.

## Data Availability

The data that support the findings of this study are available from the corresponding author upon reasonable request.
